# The life and times of Richard L. Rauck (1956–2025)

**DOI:** 10.3389/fpain.2026.1834575

**Published:** 2026-05-08

**Authors:** James C. Eisenach

**Affiliations:** Department of Anesthesiology, Wake Forest University School of Medicine, Winston-Salem, NC, United States

**Keywords:** life and times, neuromodulation, obituary, pain, rauck, spinal drug therapy

Richard Lee Rauck, “Rick” was a clinician, educator, and scientist who influenced the development of pain medicine in the US and across the world. We first met in 1986 as new faculty with adjacent offices at the Wake Forest University Bowman Gray School of Medicine, straight out of fellowships. We rapidly became collaborators, colleagues, and friends. That was Rick's way.

Rick played an important role in the creation and maturity of both academic and private practice pain clinics during their explosive growth from the mid-1980s to mid-2000s. This was a time of increased pain awareness (Campbell's “pain as the 5th vital sign”) and expansive federal and pharmaceutical funding of research leading to hope for rapid development of a slew of powerful, non-opioid analgesics. Pain clinics, once dominated by a few aging “artists” in regional anesthesia, were transformed by the advent of higher resolution imaging systems, especially ultrasound, allowing large numbers of clinicians to be trained to safely reach peripheral and central targets for pharmacologic, electrical, and neurolytic treatments. It was a time of over-reach, much hype, hidden conflicts of interest, and rapid growth of invasive therapies with poor quality evidence for efficacy that drove up reimbursement to the clinicians who applied them. Rick navigated these turbulent times with a focus on patient care, moderation, and education into the “when” and “why” as much as the “how” of invasive treatments and drug therapy. He brought these perspectives not only to education of residents and fellows but to the medical community as a whole through his leadership in organizations including the American Society of Regional Anesthesia, the International Association for the Study of Pain, the World Institute of Pain, and the North American Neuromodulation Society.

Rick was a physician scientist and engaged his fellows to participate in original research projects as part of their training. Of Rick's more than 200 publications, I collaborated on nearly a dozen, probing efficacy and mechanisms of various compounds for spinal drug therapy, sometimes first-in-human, under an NIH grant that spanned nearly 25 years. He even treated my post-spinal headache after volunteering for one of these studies with a blood patch over a holiday weekend! Rick was a prioneer in intraspinal therapy for chronic pain, leading a Phase III trial that resulted in FDA approval of epidural clonidine for intractable cancer pain, showing a survival benefit in cancer patients receiving continuous spinal morphine, and championing ethical, consensus-driven statements of chronic neuraxial therapy (the Polyanalgesic Conferences).

In 2004, Rick moved the pain clinic outside the university, establishing the Carolinas Pain Institute where, for many years, he continued in a private practice setting to train residents and fellows, perform research, and serve in leadership positions in the aforementioned national and international organizations. An avid sailor, Rick also organized a biennial continuing medical education course, “Pain in Paradise” comprised of several 8-passenger sailboats in the Caribbean, thereby adding his name to the list of adventurous sailors and pain leaders (e.g., Torsten Gordh, Narinder Rawal).

Rick was an empathic listener of his suffering patients. In speaking with some of these patients and his colleagues, I believe what Rick wrote of his teacher during fellowship, Dr. Prithvi Raj, applies equally well to Rick: “I have never had the privilege to work with a more accomplished clinician whose patients both adored him and found his bedside manner unparalleled in its compassion and insight.” (1) On the day after Rick's death, an article appeared on the front page of our local paper describing, not his academic and clinical achievements, but the passion he brought to our community for nearly 40 years, supporting multiple activities and establishing establish an annual international bicycle racing event, accompanied by a downtown festival. Rick was a friend to so many and lived his life with passion.

Rick is survived by his wife, Katie Rauck, daughters, Lauren Rauck Komanski and Catherine Lee Rauck, and grandchildren, Jack Rauck Komanski and Luke Woods Komanski. He leaves behind many communities of friends, colleagues, and collaborators. As was his way.

**Figure 1:**
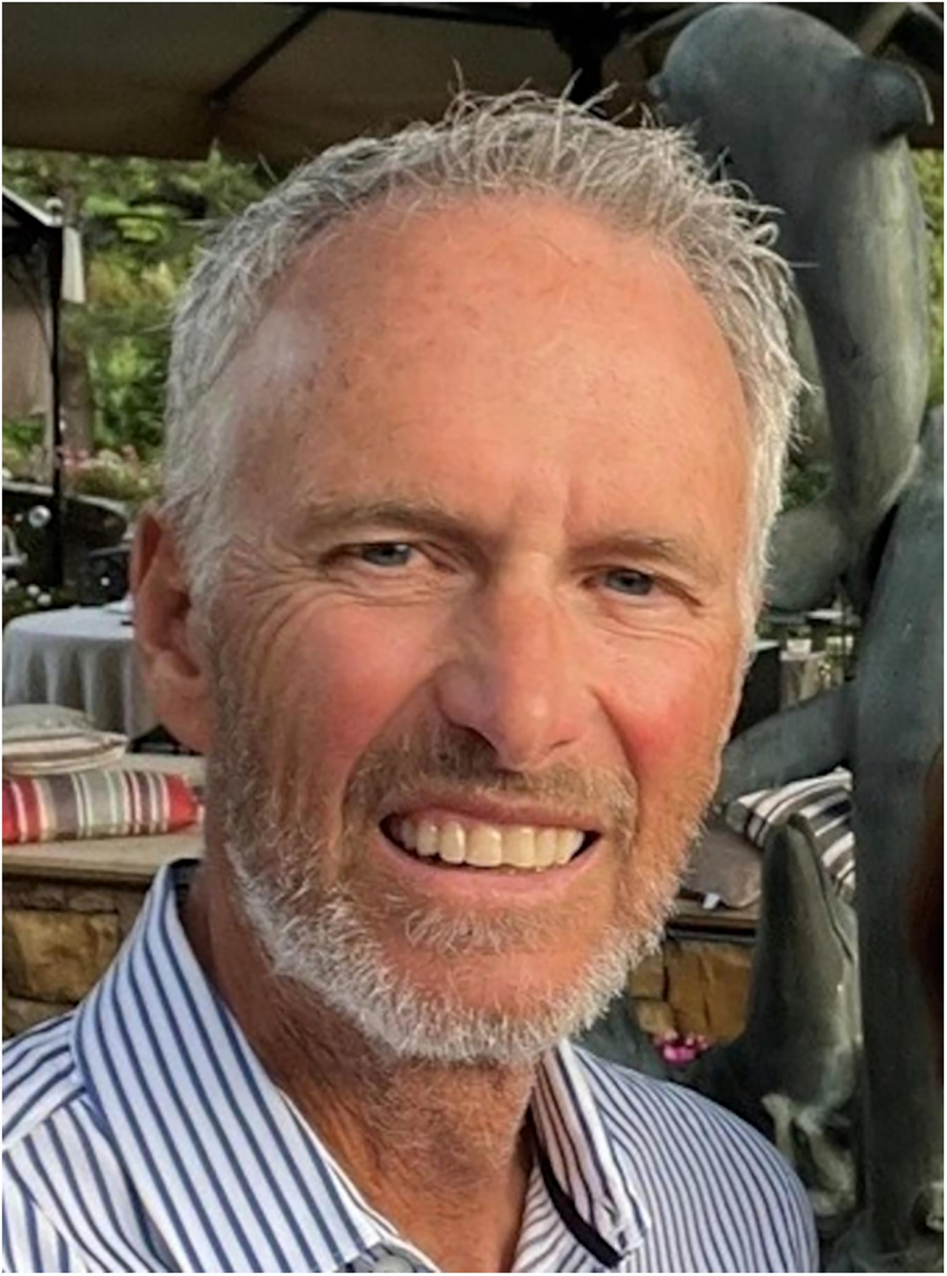
Richard L. Rauck, MD.
